# Factors affecting collaboration between general practitioners and community pharmacists: a qualitative study

**DOI:** 10.1186/1472-6963-12-188

**Published:** 2012-07-07

**Authors:** Maria Rubio-Valera, Anna Maria Jové, Carmel M Hughes, Mireia Guillen-Solà, Marta Rovira, Ana Fernández

**Affiliations:** 1 Research and Development Unit, Fundació Sant Joan de Déu, Calle Picasso 13, Sant Boi de Llobregat, Barcelona, 08830, Spain; 2 Red de Investigación en Actividades Preventivas y Promoción de la Salud (RedIAPP), Barcelona, Spain; 3 Primary Care Health Center Manso, Catalan Health Service (ICS), Carrer de Manso 19, Barcelona, 08015, Spain; 4 Clinical and Practice Research Group, Queen’s University Belfast, School of Pharmacy, Medical Biology Center, 97 Lisburn Road, Belfast, BT9 7BL, UK; 5 Primary Health Care Center Mallorca, Research Unit, Health care Services of Balearic Isles (IB-Salut), Calle Reina Esclaramunda 9, Palma de Mallorca, 07003, Spain

**Keywords:** Interprofessional Relations, Family Physicians, Pharmacists, Qualitative Research

## Abstract

**Background:**

Although general practitioners (GPs) and community pharmacists (CPs) are encouraged to collaborate, a true collaborative relationship does not exist between them. Our objective was to identify and analyze factors affecting GP-CP collaboration.

**Methods:**

This was a descriptive-exploratory qualitative study carried out in two Spanish regions: Catalonia (Barcelona) and Balearic Islands (Mallorca). Face-to-face semi-structured interviews were conducted with GPs and CPs from Barcelona and Mallorca (January 2010-February 2011). Analysis was conducted using Colaizzi’s method.

**Results:**

Thirty-seven interviews were conducted. The factors affecting the relationship were different depending on timing: 1) Before collaboration had started (prior to collaboration) and 2) Once the collaboration had been initiated (during collaboration). Prior to collaboration, four key factors were found to affect it: the perception of usefulness; the Primary Care Health Center (PCHC) manager’s interest; the professionals’ attitude; and geography and legislation. These factors were affected by economic and organizational aspects (i.e. resources or PCHC management styles) and by professionals’ opinions and beliefs (i.e. perception of the existence of a public-private conflict). During collaboration, the achievement of objectives and the changes in the PCHC management were the key factors influencing continued collaboration. The most relevant differences between regions were due to the existence of privately-managed PCHCs in Barcelona that facilitated the implementation of collaboration. In comparison with the group with experience in collaboration, some professionals without experience reported a skeptical attitude towards it, reporting that it might not be necessary.

**Conclusions:**

Factors related to economic issues, management and practitioners’ attitudes and perceptions might be crucial for triggering collaboration. Interventions and strategies derived from these identified factors could be applied to achieve multidisciplinary collaboration.

## Background

General practitioners (GPs) and community pharmacists (CPs) are encouraged to collaborate to improve patient care [1,2] Pharmacists’ interventions within the healthcare team improve patient outcomes in physical [3-5] and mental conditions [6]. On the other hand, miscommunication between GPs and CPs is a cause of preventable hospital admissions [7]. However, during the implementation of a trial evaluating a complex intervention [8] we realized that GPs and CPs had difficulties communicating with each other. Despite working in the same geographical area and sharing patients, some doctors and pharmacists used the study researcher to transmit information to the other professional or to obtain additional information about the participants. Surveys have been conducted exploring this issue, pointing out that exchange characteristics (i.e. trustworthiness or role specification) are the factors most frequently associated with GP-CP collaboration [9,10] but quantitative work provides only a limited understanding of what promotes collaboration. Qualitative studies may untangle some of the deeper reasons preventing such collaboration [11]. Qualitative work has been conducted in the UK, Australia and USA to explore factors affecting GP-CP collaboration [12-15]. These qualitative works highlighted the fact that professionals from each discipline were not personally acquainted, territoriality, and the pharmacist’s conflict of interest with regard to selling medications as the barriers that most affected mutual trust and respect between practitioners, thus impeding collaboration. Some of these studies explored the factors affecting collaboration in areas where multiple chain pharmacies and single independent pharmacies coexisted, reporting higher distrust and lack of interest among GPs towards collaboration with chain pharmacists. However, the impact on collaboration of publicly funded and privately managed PCHC in comparison with publicly funded and publicly managed PCHCs has not been previously assessed. Nor did these studies explore the perception of the Primary Care Health Centers (PCHC) managers or the impact on the GP-CP relationship of external agents that participate in the process such as the patient and the pharmacy assistant.

Differences between the health systems and the model of community pharmacy require a country-specific study in Spain. The Spanish national health system (NHS) is publicly funded. The organization and provision of health services depends on each of the 17 regional governments through which Spain is governed [16]. This generates differences in health policies between regions [17,18] which could affect the organization of primary care health centers and pharmacies as well as the way in which collaboration is manifested between GPs and CPs. Table 1 summarizes the main characteristics of Spanish primary care, comparing the organization of the PCHCs and the pharmacies.

**Table 1:** Summary of the main characteristics of the PCHC and community pharmacies in Spain

	**Primary care health centers**	**Community pharmacies**
Owner	Predominantly state-owned	Privately owned (the pharmacy owner must be a licensed pharmacist and each pharmacy may own only one pharmacy)
	Every PCHC contains several GP surgeries with few exceptions in rural areas of Spain	
Funding	Publicly funded	Offers both publicly funded services (i.e. drugs that are financed by the state) and privately funded services (i.e. over the counter drugs)
		A large part of the profit derives from selling financed drugs.
Management	Predominantly publicly run (the manager is one of the GPs from the PCHC team that combines clinical activities with management activities)	Privately run (usually by the owner)
	In some regions, privately managed PCHCs exist. This is the case with the "Entitades de Base Asociativa" (EBAs). EBAs are limited companies comprised of health professionals that establish a contractual relationship with the NHS to offer health services in exchange for capitation financing, a theoretical cost per person independent of the real costs incurred.	
Compensation	Most GPs are employed by the public sector and receive fixed salaries.	CPs are owners of the community pharmacy or employed in exchange of a fixed salary.
	Management by Objectives (MBO) has been introduced to improve quality of the service and reduce cost. For instance, GPs are paid a bonus if they prescribe a high percentage of generic drugs and/or those of proven efficacy.	

The aims of this study were: 1) to identify and analyse barriers and facilitators in collaboration between GPs and CPs in Spain and 2) to explore whether differences exist between GPs and CPs based on the geographical region where they work and previous experience of collaboration.

## Methods

A descriptive-exploratory qualitative study using face-to-face, semi-structured interviews (January 2010-February 2011) was undertaken using a phenomenological approach.

The study population comprised GPs and CPs from 2 Spanish regions: Catalonia (Barcelona) and the Balearic Islands (Mallorca). Commitment to primary care is higher in Catalonia than in Balearic Islands and there is a greater investment of resources in primary care with respect to total health expenditure [18] Consequently, in comparison with Balearic Islands, there is a higher density of PCHCs, a lower ratio of patients per GP, a greater number of services integrated within primary care (i.e. dentistry) and better access to diagnostic procedures by GPs in Catalonia. A further important difference between the regions is the coexistence of the private and public model in Catalonia, which is reflected in the presence of "Entidades de Base Asociativa” (EBAs) (see Table 1) that do not exist in the Balearic Islands. In both regions management by objectives (MBO) is used. The MBO system gives GP incentives (usually financial) if they fulfill the objectives set by the health system (i.e. prescription of drugs of proven efficacy). Electronic prescribing links the GP’s prescriptions with the community pharmacists and acts as a communication channel between the two professionals. The implementation of electronic prescribing in the Balearic Islands began in 2006, long before its introduction in Catalonia. By the time we conducted the study interviews, electronic prescription had long been established in the Balearic Islands while in Catalonia only GPs and CPs from some areas were using it, primarily in rural areas and small cities. In Barcelona, it was in the implementation stage.

We also considered whether recruited practitioners had previous experience of collaboration (defined as having had regular face-to-face contact with the other group of professionals). We theorized that practitioners from different regions and those with previous experience compared with those with none would have different opinions towards collaboration thus maximizing the possibility of finding disconfirming cases (theoretical sampling [11]).

The PCHC manager in Spain is usually a GP from the team of physicians who combines clinical work with the management of the PCHC. While in charge, the manager officially represents the PCHC and he/she administers the human and financial resources allocated to the health center. Consequently, the PCHC manager is responsible for distribution of the resources necessary for collaboration with the CP. Some of the GPs interviewed had experience as managers of the PCHC.

We contacted key informants from the fields of primary care (PC) and community pharmacy in Barcelona and Mallorca [the College of CPs, the Research Network on Preventative Activities and Health Promotion (RedIAPP) in PC and the School of Pharmacy-University of Barcelona] to identify those professionals who matched our sampling criteria. It was explained to the key informants that we were seeking professionals with experience of collaboration so that we would be referred to professionals whom we knew in advance would take part. Thereafter, practitioners were contacted by telephone and invited to participate. At this time we established a time for the interview but did not explain the study objective in detail. We did, however, ask them some brief questions, including whether they collaborated with any other health professionals. Once interviewed, participants were asked if they knew other practitioners who matched our criteria (snowball sampling [19]). One general practitioner declined to participate.

Participants were recruited until each theorized category (professional group, previous experience and region) independently achieved saturation of thematic findings.

Interviews were conducted in a place of convenience for the participant by AMJ (GPs from Barcelona), MRV (CPs from Barcelona), MG (GPs from Mallorca) and MR (CPs from Mallorca). The interview guide was developed by a team of researchers and clinicians including AMJ, AF and MRV taking into account their experience in the field as well as the results of the only paper that had been published at the time the study was designed [13]. The interview guide was piloted with one GP and one CP. The interview guide is summarized in Table 2. Interviews were audiorecorded, fully transcribed and anonymized.

**Table 2:** Topic guide for the interview

Topic guide	Suggested questions to help the interviewer
Relationship nowadays	*How is your relationship with the CP/GP?*
	- If there is no relationship: *Why do you think that there is no relationship?*
	- If the relationship is good/bad/regular: *What do you think that makes the relationship good/bad/regular?*
Utility of the collaboration	*Do you think that it would be useful to potentiate the teamwork between the GP and CP? Why?*
	*What advantages do you see in working in collaboration with the CP/GP?* *And what disadvantages do you see in collaborating with the CP/GP?*
Opinion about the other group of professionals	*What do you think about the CP/GP?*
	*How do you think that CP/GPs see GP/CPs?*
Barriers for communication	*If you tried to get in contact with the CP/GP at any time, what difficulties did you have?*
	*You told me that when trying to get in contact with the CP/GP you had problems because… can you think of any others?*
Barriers for collaboration	*What do you think makes collaborative work difficult?*
Facilitators for communication	*What steps do you think could be taken to improve communication with CPs/GPs?*
Facilitators for collaboration	*How do you think collaborative work could be promoted or strengthened?*
Impact from the National Health System	*Is there any aspect in the organization of the health system that you think is affecting the relationship between GPs and CPs? In what sense?*

GP = General practitioner; CP = Community pharmacist.

In parallel with interviews, analysis assisted by Atlas-ti software was conducted. The information obtained was triangulated by the participation of three investigators [a GP (AMJ), a pharmacist (MRV) and a psychologist with experience in using qualitative research (AF)] who independently analyzed the interviews [11]. In the Mallorca interviews, a fourth analyst participated [a nurse and sociologist (MG) from Mallorca].

Analysis was conducted using Colaizzi’s method for analysis [20]. The following is a complete description of the procedure. The process of generation of categories was largely inductive. Researchers became familiar with the interviews by listening, reading and re-reading them. Themes were identified and coded independently by each of the researchers involved in the analyses. Researchers then came together to compare and discuss differences in the analyses. Themes were then re-coded and classified, identifying common patterns and convergences and divergences in data through a process of constant comparison. With the assistance of a fifth researcher, a pharmacist with experience in undertaking qualitative research and collaboration between pharmacists and GPs (CMH), findings from the analysis were integrated to formulate a theoretical model for the phenomenon under investigation. Finally, respondent validation was conducted, comparing our interpretation of the phenomenon with those who had participated. Participants were sent a summary of the findings and invited to a meeting where findings were presented and discussed. Fourteen out of thirty-seven professionals participated in this validation. Changes suggested by participants were incorporated into the final description of the phenomenon.

In order to guarantee the validity of this research [11,21] the study was externally audited from the beginning to its conclusion by a group of researchers from the "Qualitative Health Research Group" (led by Dr Vázquez ML) of the "Consorci de Salut i Social de Catalunya". Interviewers and main analysts kept a personal research diary in which any reactions to events occurring during the research were recorded. All participants gave informed consent and the study was approved by the Foundation Sant Joan de Déu Clinical Research Ethics Committee.

## Results

A total of 37 interviews were conducted that lasted 5–99 minutes (mean: 23 minutes) (Table 3 shows the sample characteristics). Nine GPs and nine CPs had had previous experience in collaboration with the other group of professionals and 4 of the GPs belonged to an EBA.

**Table 3:** Characteristics of the participants

**Characteristics of participants *(n = 37)***	**Barcelona**	**Mallorca**
**General practitioners,*n***	9	9
Sex, *n*		
Male	6	4
Female	3	5
Mean age (range), years	48.7 (35–60)	47.6 (37–60)
Mean clinical work experience (range)*, years	21.8 (8–31)	19.6 (8–30)
Previous experience in GP-CP collaboration, *n*		
With previous experience	4	5
Without previous experience	5	4
**Community pharmacists,*n***	10	9
Sex, *n*		
Male	4	4
Female	6	5
Mean age (range), years	50.4 (30–64)	47.7 (29–56)
Mean clinical work experience (range), years	23.7 (5–40)	17.2 (1–30)
Previous experience in GP-CP collaboration, *n*		
With previous experience	5	4
Without previous experience	5	5

* Three general practitioners from Barcelona and four from Mallorca also had experience as Primary Care Health Center managers.

In the group of participants with previous experience the collaborative experiences differed from one another. Some examples of activities were: interdisciplinary professional training, detection and resolution of medication related problems, carrying out tests or clinical analysis (e.g., glycemia) at the pharmacy for patients who require regular monitoring, rationalization of expenses, special care for people with mobility problems, personalized medication dosage system (weekly blister packs for patients taking a variety of medications) and public health education, and so on.

### Factors affecting GP-CP collaboration

The factors affecting the relationship varied depending on the timing in relation to the collaboration: 1) Before collaboration started (prior to collaboration) and 2) Once the collaboration had been initiated (during collaboration).

Prior to collaboration, GPs and CPs worked on their own. This first phase was a process of team-building that allowed collaboration to begin. The factors identified assigned to this first stage in the process of generation of the theoretical model were central in facilitating or impeding the initiation of collaboration between the two professions.

During collaboration, a successful relationship between doctors and pharmacists has been established and the factors affecting that relationship changed. The challenge from this point on is to maintain and consolidate collaboration to ensure continuity.

Practitioners from the group which had experience of collaboration provided data which was rich in the identification of collaboration facilitators. GPs and CPs without experience who had tried to collaborate without success provided information about barriers which prevented or limited collaboration. Finally, GPs and CPs without experience who had never engaged in collaboration reported barriers related to attitudes and preconceived perceptions.

### Prior to collaboration

**Figure 1:**
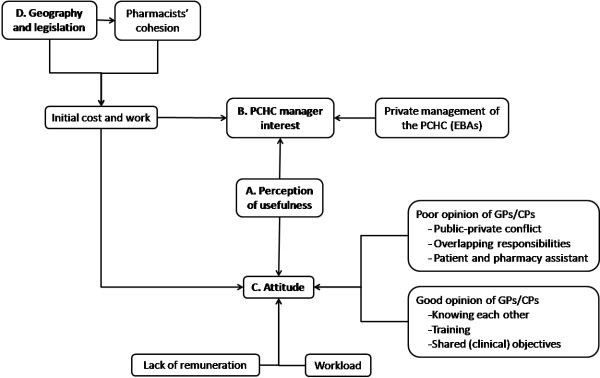
**Factors affecting collaboration between general practitioners and community pharmacists prior to collaboration.** PCHC: Primary Care Health Center; EBA: type of privately managed PCHC; GPs: General practitioners; CPs: Community pharmacists.

Prior to the process 4 key factors were identified which affected collaboration: A) perception of usefulness, B) PCHC manager’s interest, C) attitude, and D) geography and legislation. Figure 1 shows the conceptual model of factors affecting the initiation of the collaborative relationship between GP and CP.

#### Perception of usefulness

A positive perception of usefulness was necessary in order to start the collaboration. Conversely, the perception of usefulness was negative when GPs and CPs believed that there were no advantages in collaborating or that collaboration would cause problems. Only professionals without previous experience from Barcelona thought that collaboration would be troublesome.

"I think we shouldn’t tamper with it (the relationship with the doctor) because I think that it’s correct … if we look for something more we will have problems. [CP2: Community pharmacist without experience in collaboration from Barcelona (CP WO BCN)]."

However, participants stated that there were some factors that could influence a positive perception of usefulness. GPs and CPs with previous experience reported that evidence supporting positive outcomes of collaborative GP-CP relationships could make professionals change their mind about collaboration. Similarly, professionals stated that sometimes the NHS introduced strategies that affected both groups of professionals, e.g., the introduction of electronic prescribing, which could force a collaboration to start.

"But thanks to electronic prescribing, given that just like any implementation of a system in which we are forced to work together … has forced this exchange, this feedback with the medical team … we have established a series of courses of action with the aim of having more fluid communication to solve this problem. [CP10: Community pharmacist with experience in collaboration from Barcelona (CP W BCN)]"

#### PCHC manager interest

To collaborate it was necessary that the PCHC manager was interested in promoting collaboration.

"I think that a lot depends on the will of the manager of the PCHC; whether the manager of the PCHC is in favor or not. So far I have had a PCHC manager against (cooperation). Now they have changed this and she is already waiting for me. [CP1: CP WO BCN]"

The interest of the PCHC manager was influenced by his/her own perception of usefulness and by the initial cost in terms of infrastructure and human resources required to trigger the collaboration. EBA-type PCHCs in Barcelona were a good example of this (see Table 1). GPs and CPs suggested that collaboration was easier for two reasons: because the PCHC manager would be interested in collaboration as a strategy to reduce costs and improve outcomes for the center and because these PCHCs had smaller teams which were easier to coordinate.

"The advantage of centers like this (EBA) is that this health center is a small center. … There is more flexibility and greater speed when we want to get a project going. [GP8: General Practitioner with experience in collaboration from Barcelona (GP W BCN)]."

A barrier influencing the manager’s interest was the perception that the NHS did not incentivize collaboration.

#### Attitude of the professionals

Attitude was strongly influenced by opinions held about the other professional. A good opinion would lead to respect and trust; key factors for collaboration. A negative opinion might be due to the perception that a “public-private” conflict existed. GPs and CPs believed that, through MBO (see Table 1) doctors were encouraged to prescribe cheaper drugs and less of them while the pharmacist, through selling medications, had a greater interest in non-rational use of medicines.

"(Pharmacists must think) that we are forced by the health policies that reward or punish some prescription styles … There are many doctors that (say) “I’m not giving (prescribing) this, do you know why? Because I’ll get into trouble, because they’ll penalize me”… They (CP) must think, “Here, it’s my money that’s at stake, because I have a business and the doctor is a state employee and nothing’s going to happen to him/her and he/she doesn’t care.”… And they must compare this difference of their feeling of responsibility, that they have a business and they must pay a salary to their assistants, that there are things to pay for. They have an element of the entrepreneur that we don’t have. [GP8: GP W BCN]"

Overlapping responsibilities generated a negative opinion of the other professional. GPs and CPs believed that sometimes the other professional was performing tasks that should not be done by him/her. This concern was generated by the fact that the roles of professionals were not well defined.

"We have the experience of seeing a productive cough, a dry cough, some mucosity. I think we can recommend a medication … a doctor will tell you: “but well, we are doctors and it is us who have to (prescribe)” and, well, he/she is right, on the one hand they’re right, but where is the line where we end and the doctor begins, you know? [CP7: CP WO BCN]"

GPs and CPs believed that patients generated conflict by relaying biased information or directly criticizing professionals.

"(The patient) is a tell-tale (laughs). Sometimes they also tell lies, you know? … They tell lies to both groups (the doctors and the pharmacists). This is also true, eh? They are blackmailers, they blackmail to get what they want. [CP6: CP W BCN]"

Pharmacy assistants created conflict by assuming roles of the pharmacist. This affected quality of patient care and the relationship with the doctor, who preferred to communicate with the pharmacist.

"For some years, anybody could start off being the pharmacy guy (pharmacy assistant) … He then started dispensing and ends up putting on a white coat and finally he acts as a pharmacist … the boss, the licensed pharmacist, he is almost always there but if he/she is not there, and I need to talk about something and I need his/her knowledge [GP8: GP W BCN]"

However, the view about the other profession changed when practitioners knew each other. Stigmatized views and conflicts were resolved.

"The main advantage when collaboration is established is that it breaks a series of stereotypes that exist from the doctor towards the pharmacist, that there is intrusiveness, this or that… and the opposite, from the pharmacist towards the doctors, that they are arrogant, that they do this or that, all these things stop when two professionals with similar knowledge, or even a similar age, see each other, a lot of barriers are broken. [CP16: Community pharmacists with experience from Mallorca (CP W MLL)]"

Another factor that may contribute to improving the physician’s opinion of the pharmacist is the existence of shared goals to improve service, preferably if they are clinical. Put bluntly, GPs and CPs felt that collaboration was only possible if the pharmacists involved in the team were highly trained and clinically competent.

Initiating collaboration involved extra work for these busy professionals who received no additional remuneration for it. GPs and CPs stated that their attitude would be even worse if the NHS forced them to collaborate without releasing them from other duties or offered economic incentives.

"To attend to a patient, time is needed and this time is also money. And trained people are needed. For training, time and money are needed, you see? Everything until now has been paid by the pharmacist him/herself. … If fewer human resources are available, then somewhere we will have to make cuts. [CP10: CP W BCN]"

#### Geography and legislation

Legislative and geographical factors had the potential to increase the effort required to coordinate collaboration, which in turn also affected the professionals’ attitudes. In poorly defined geographic areas with no clear neighborhood divisions, there were a large number of pharmacies and/or some of them were far from the PCHC. By law, patients can choose any pharmacy to fill their prescription and can switch from one to another in successive visits, making collaboration difficult. Therefore, professionals felt that working together was easier in small areas or when pharmacists in the area worked together and coordinated the delivery of services between them.

"What happens is that I have the advantage that I am in a basic unit where I have a single reference pharmacy. So, of course, there is only one pharmacy with which I have regular contact … which is a very big advantage. [GP17: General practitioner with experience from Mallorca (GP W MLL)]"

According to GPs and CPs, another barrier is caused because pharmacies are privately owned and pharmacists are not seen as part of the health system structure.

"I consider that the structure clearly leaves the pharmacist outside the national health system. That’s why the pharmacists don’t know which entity they are part of. We are private centers with an agreement with the administration. Yes, we are obliged to follow all the administration guidelines … because we are dispensing national health system prescriptions but then we are not considered as being part of the health system in any way. Not structurally, organizationally, legally, nowhere. If one is not considered (as part of the system) it is very difficult to be part of it. [CP16: CP W MLL]"

In the current system, pharmacy income is mainly centered on the payment for dispensing medications. Pharmaceutical care services which do not involve the sale of medications are, therefore, not remunerated so that doctors and pharmacists think that the system promotes an economic conflict of interest that makes it difficult for the pharmacist to collaborate on clinical tasks.

"What we want is what is right for the patient. And, of course, earn our living with the medications … if only they paid me differently. That is a problem. I mean, I think that a problem that our relationship could have (the doctor with the pharmacist) is the idea that the doctor has that the pharmacist makes a profit from the medication. And I think that, well, we are earning a living, of course, and at the moment we earn a living with medications. It’s a handicap we have. [CP4: CP WO BCN]"

Some doctors and pharmacists suggest that one possible solution is a change in the organization of the pharmacy, integrating it into the health center itself.

"One possible solution which is not totally unviable is to create pharmacies inside the health center. So that there is a pharmacy service like there is in the hospitals. … De-privatize the pharmacies. [GP17: GP W MLL]"

### During collaboration

**Figure 2:**
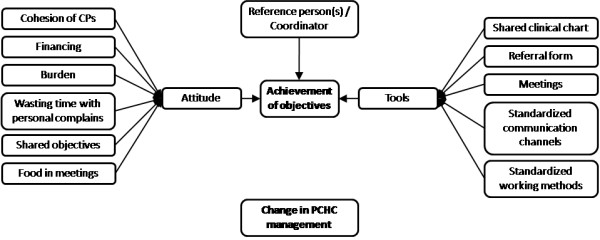
**Factors affecting collaboration between general practitioners and community pharmacists once the collaboration has been started.** PCHC: Primary Care Health Center; CPs: Community pharmacists.

Once collaboration had successfully started, the factors that influenced the relationship changed. Two key factors were identified at this stage: A) achievement of objectives and, B) change in the PCHC management. Figure 2 shows the relationship between the factors affecting GP-CP collaboration once collaboration had begun.

### Achievement of objectives

For the relationship to be maintained over time, both professionals and the PCHC manager had to recognize the benefit of this collaboration. There were a number of factors that could help to fulfill the goals. According to GPs and CPs, it seemed essential to have a coordinator(s) or reference person(s), responsible for leading the collaboration and linking the two professional groups.

GPs and CPs felt that meetings needed to be held regularly so that professionals could discuss shared objectives. Meetings between GPs and CPs usually take place in the PCHC at lunch time, when the pharmacies are usually closed and the GPs change shifts so when food was provided it encouraged attendance. While this is not an ideal arrangement, the option of combining lunch with meetings is the most practical solution in a country where the midday meal is a social occasion and many business premises, including pharmacies, close between 2-5 pm. It was important to share a clinical chart so that both professionals could have access to complete patient information. Standardized working methods as well as standardized communication channels facilitated collaboration. For non-urgent consultations, a referral form assisted in information transmission.

"One of the things that we had is that we phoned the health center to have direct access to the doctor’s surgery. We had the switchboard number,… each doctor had a switchboard number and we dialled it. I mean we went in directly. This meant that any problem we had could be solved straight away. [CP6: CP W BCN]"

However, a positive attitude might change as a consequence of the increased burden or lack of financing that made collaboration impossible.

"As we already have a lot of work, it couldn’t work and it was cancelled. Because we all have enough work … I don’t think there was any other reason, there was no misunderstanding or anything else. It was just this, the pressure they were under and ours too. [GP4: GP W BCN]"

In addition to workload, wasting time with personal complaints in meetings could make GPs and CPs unwilling to collaborate. Pharmacists needed to work with each other and could be demotivated if they were unable to cooperate with their colleagues. Finally, if GPs and CPs were unable to agree on new objectives, collaboration would end.

"Sometimes we (GPs and CPs) have meetings in the health center and the differences (between GPs’ and CPs’ interests) are so divergent that we don’t have points in common. I mean, they (CPs) have their interests, that if their stock, that if I don’t know what … When we are having these meetings and you say: “but if we (GPs) really don’t mind that the stock of generics is this brand or another”… Sometimes they speak about things that we don’t understand [GP13: General practitioner without experience from Mallorca (GP WO MLL)]"

### Change in PCHC management

If the new management team was not in favor of collaboration, practitioners would no longer have time or support to conduct meetings, collaborative work, etc.

"The most important handicap appeared when they (EBA’s managers) left… No other company was contracted; they (the people of the NHS) decided to manage the center by themselves. They designated a new manager and, at the beginning, we explained to her everything we had been doing and everything looked fine to her “very good, very good, very good” but we had neither meetings, nor health controls, nor… I mean, everything diminished. [CP7: CP W BCN]"

## Discussion

### Summary of main findings

This study highlights two stages associated with collaboration: prior to and during collaboration. Key factors prior to collaboration were perception of usefulness, PCHC manager interest, attitude, and geography and legislation. At this stage there was a process of team building that corresponded to the first stages of the model of development of Collaborative Working Relationship (CWR) [22] (Professional Awareness; Professional recognition; Exploration and Trial). During collaboration, which corresponded to the last stages (Professional expansion; Commitment to the CWR), achievement of common objectives and PCHC management stability were the main factors to consider in perpetuating collaboration.

The most important difference between regions in terms of collaboration was due to the presence of EBAs, which only exist in Barcelona. Practitioners from this type of privately managed center were more motivated to initiate collaboration with the community pharmacies and, once initiated, the relationship seemed to be easily maintained over time.

Negative perception of usefulness was only reported by GPs and CPs without experience in collaboration from Barcelona. The fact that this particular view was only reported by professionals without previous experience in collaboration could be a consequence of previous bad experiences when having contact with the other professional. Although we consider that it could be the other way around and, in fact, it is the negative perception, or prejudice against collaboration, that prevents it. Regional differences cannot be explained with the information we have at present. It is possible that the search for negative cases (i.e. disconfirming cases) was more intensive in Barcelona than in Mallorca although it is also possible that the variance really is due to regional differences. This issue will require further exploration.

GP and CP speech were similar and they agreed on the majority of factors affecting collaboration. Although there are differences in the factors which affect each type of professional (i.e., the pharmacist’s conflict of interest is related to an incentive to sell the greatest number of products while that of the doctor is connected with offering a service in which cutting costs results in salary bonuses) both professionals express them, being aware that this is the view others have of them which, in turn, affects collaboration.

### Strengths and limitations

To the best of our knowledge, this is the first qualitative study conducted by a multidisciplinary team and the first conducted in Spain. Moreover, this is the first study that compares samples from two regions with different geographical characteristics and health policies. Taking into account distinct contexts improves the relevance of this study. A series of quality control measures were used to guarantee the trustworthiness of the conclusions.

Nevertheless, those who participated in the study were more likely to be interested in this topic. However, we recruited professionals without experience in collaboration and who held negative views regarding collaboration.

Interviews were conducted by a number of investigators, perhaps resulting in biased information, but an interview guide was used and recorded interviews were audited. However, CPs were interviewed by a pharmacist, and GPs were interviewed by a GP or a nurse, encouraging openness and honesty.

### Comparison with existing literature

Results from this study are consistent with previous research [12-15]. The importance of mutual knowledge, role definition, CPs’ conflict of interest and the territoriality of the GP concurs with previously reported results [12-15] although in our study a problem with territoriality was also reported by pharmacists who felt that GPs were assuming pharmacist roles. Since some GPs in Spain are incentivized through MBO, a stereotypical view of the GP being too worried about meeting targets and being rewarded by the health system was also demonstrated in our study.

Both professions had to perceive collaboration as economically profitable. However, as highlighted by our study, the PCHC manager also had to be motivated to promote this collaboration. In privately managed PCHCs, practitioners stated that collaborative care had led to a reduction in expenses, which could have been a motivation for managers to collaborate. This could be an important factor to consider when implementing collaborative relationship in areas or countries where PCHCs are privately managed.

Pharmacists in Spain only receive public funding for providing prescription medicines. Consequently, barriers related to lack of incentives from the NHS to initiate and maintain collaborative work were highlighted. Previous studies have reported pharmacists’ concerns about potential increases in workload and adequacy of remuneration when new services are introduced [23] seemed to be a crucial factor in building multidisciplinary teams [12,14,15]. When professionals meet, preconceptions about the others can be overcome and shared aims, strategies and tools to enhance communication and lead to an improvement in services can be discussed. To maintain collaboration, it is preferable to share clinical objectives [14]. If only administrative issues are addressed, physicians are not interested and pharmacists feel frustrated. Working on clinical issues implies sharing patient clinical information [14]. Ethical and security considerations need to be taken into account when collaborating and the need for patient consent must be considered [22].

## Conclusions

A better understanding of the GP-CP relationship enables us to develop strategies and interventions to promote collaboration. The most relevant strategies to implement are the encouragement of positive attitudes and the perception of usefulness on the part of the health administrators and professionals to take advantage of the new changes or strategies imposed by the health system as an opportunity to initiate collaboration (common objective); to promote face-to-face relationship development to overcome prejudices and enable team work initiation and development; to designate coordinators responsible for coordinating teamwork; and to establish standardized agreed communication. Future research needs to be conducted to evaluate the effectiveness of these strategies.

## Competing interests

The authors declare no competing interests.

## Authors’ contributions

MRV and AF developed the original idea for the research and, together with AMJ, designed the study protocol and coordinated the study. AMJ, MRV, MG and MR conducted the interviews. AMJ, MRV, MG, AF and CH participated in the analysis. MRV drafted the manuscript with the participation of AF and AMJ. All authors have corrected draft versions and approved the final version of the manuscript.

## Pre-publication history

The pre-publication history for this paper can be accessed here:

http://www.biomedcentral.com/1472-6963/12/188/prepub
